# Bundesliga team values: Deciphering the impact of performance and economics

**DOI:** 10.1371/journal.pone.0312810

**Published:** 2024-12-02

**Authors:** Efehan Ulas, Christian Deutscher

**Affiliations:** 1 Biostatistics and Medical Informatics, Kirklareli University, Kirklareli, Turkey; 2 Sport Economics, Bielefeld University, Bielefeld, Germany; Privatuniversität Schloss Seeburg: Privatuniversitat Schloss Seeburg, AUSTRIA

## Abstract

Despite experiencing a dip during the COVID-19 pandemic, football remains a robust multi-billion-euro industry. The accurate prediction of team values holds immense significance for teams, investors, and other stakeholders. In this research, we delve into the determinants of German Bundesliga team values, encompassing performance-based metrics, macroeconomic indicators, and demographic statistics. Leveraging machine learning and dynamical linear methods, we construct a comprehensive model for Bundesliga team values. Our findings not only illuminate team performance on and off the field but also offer vital insights to guide decisions and navigate the complexities of the fiercely competitive football industry.

## Introduction

Football has flourished into a thriving industry, attracting immense investments. Its captivating allure has turned football into a global entertainment industry and propelled it to an astonishing economic scale, global football revenues surpass 1 trillion euros annually, with significant impacts on the global economy and employment. Identifying the key factors of team values holds tremendous potential in shaping the strategies employed by football clubs. In the 2023-2024 season, the German Bundesliga solidified its position among UEFA countries, securing a commendable fourth place with an aggregated team value of €4.43 billion (transfermarkt.com). Leading the rankings, the English Premier League takes the premier spot with an astounding team value of €11.27 billion, followed by the Spanish La Liga at €5.16 billion and the Italian Serie A at €4.79 billion (transfermarkt.com).

This paper disentangles the determinants of team values in the German Bundesliga. It analyses the drivers of team values and identifies common features and characteristics among teams to refine and optimize the model structure using dynamic linear modelling techniques. We utilize the values of German Bundesliga teams over the past 13 seasons, including research-based sporting determinants such as historic sporting success. To achieve a nuanced and accurate model, it becomes imperative to integrate the demographic and economic parameters of the cities associated with the teams. This approach reveals the intricate relationship between team values and sporting achievements. The result is an efficient and adaptable model that captures the dynamic nature of team values in the German Bundesliga, providing valuable insights into their trends and patterns over time.

## Literature

As a factual player and team values cannot be measured, literature relies on information based on the wisdom of the crowd who evaluates them on the basis of their expertise. As we operationalize team values as the sum of individual player values, we do assume that the individual player value is detached from the identity of the team (and the players teammates). It is important to note that the player values reported by www.transfermarkt.com are expected player values in a free market. Such values are not necessarily identical to actual transfer fees. While the concept has been critiqued by Ackermann [1], research reports a strong correlation between the player evaluation on www.transfermarkt.com and materialized transfer fees. (e.g. [2, 3]).

As this paper tackles the drivers of team values, is contributes to multiple strands of literature. First, our paper relates to work that covers the determinants of individual player values. [4] explores the impact of job mobility on the player’s market value. Accordingly, market observers assess player values with imperfect information and utilize job mobility as a signal to gauge the qualities, skills, and playing potential of footballers. In line with agency theory and information economics, [5, 6] affirm that players have private information about their ability and show that players self-select into clubs and that such self-selection is a signal for player quality. As players have private information, teams rely on various signals to evaluate player values. Here, managers obtain pertinent information during the player evaluation process [7]. Signals particularly cover player performance indicators which are sorted against the position and hence positions specific tasks required from the player [7]. As requirements for a position vary between leagues, the determinants for player’s transfer fees also do vary [8].

As we sum individual player values to team values, this paper secondly suits the literature on team value analysis. Respective research across various sports is extensive, but the specific focus on football remains limited due to the complex nature of the sport. Outside of soccer, studies feature various sets of team value drivers. [9] emphasizes that a team’s genuine value in sports arises from the collective impact of coaches, fans, and players on both financial prosperity and competitive success. [10] assess show that franchise longevity, historical performance, market dynamics, and efficiency contribute significantly to the value of professional sports franchises [11] investigate the economic ramifications of prosperous NFL franchises and also highlight economic prosperity accompanying sporting success, especially Super Bowl victory. [12] add that team franchise values are positively influenced by market size, team performance, and the availability of a modern facility. They observe that the utilization of a regional identity contributes to franchise values differently across major leagues; making an argument for league specificities in line with [8]. In similar fashion, [13] find different correlations between the ownership status of playing facilities and franchise across major league sports. Exceptionally covering European football, [14, 15] determine team values in European soccer to reveal that player valuations, clubs’ operating income, and new ownership variables impact club valuations, alongside factors such as stadium age, club ownership type, supporter numbers and income, as well as past sporting performances. Yet, models that merely emphasize team performance and success criteria in team market value fall short of providing a comprehensive understanding. Research has uncovered a correlation between a nation’s or region’s income and population and its success in football [16, 17]. Countries with higher national incomes and larger populations tend to achieve more success in the sport [18–21]. To achieve a nuanced and accurate model, it becomes imperative to integrate the demographic and economic parameters of the cities associated with the teams. This approach reveals the intricate relationship between team values and sporting achievements. While the literature has explored team values in various sports branches, it is surprising to find a lack of research specifically modelling or analyzing the values of Bundesliga teams—a gap this papers attempts to fill.

## Materials and methods

### Data

For Bundesliga seasons 2010/2011 to 2022/2023, our research endeavour focuses on the meticulous collection of a diverse array of data pertaining to sixteen esteemed teams. It encompassed key economic aspects such as the dependent variable team value, the sum of the player salaries or TV revenue. It displays the team’s sporting accomplishments and roster information, such as the average age of the players. To ensure the accuracy and reliability of our data, we conducted exhaustive data scraping from reputable sources, most notably the websites www.transfermarkt.com and www.capology.com. These platforms, known for their proactive approach to sourcing valid and trustworthy team market values, performance statistics, and total salary, are proven resources for academic research [2, 22]. For economic and demographic information of the cities and states associated with the esteemed teams, we rely on official sources such as www.statistik.arbeitsagentur.de, www.ceicdata.com, and www.macrotrends.net. Where needed, missing information was drawn from the annual reports of the municipalities.

Intriguingly, we encountered certain challenges in our quest for up-to-date economic and demographic data. However, we handled this challenge by leveraging the annual reports of the municipalities to which these cities and states were affiliated. By integrating this supplementary information, we ensured the comprehensiveness of our analysis.

### Measures

The data encompasses 16 German Bundesliga teams for the 2010/2011–2021/22 seasons, comprising 15 independent variables and one dependent variable. These variables collectively encompass a spectrum of factors, including economic indicators, demographic statistics, and team performance metrics. It is important to note that these variables and performance indicators pertain to the aggregate values of the teams as entities rather than the specific attributes of individual players. The objective of our analysis rests upon the dependent variable, denominated as “Team_Value”. Specifically, this dependent variable pertains to the market values attributed to the Bundesliga teams in a given season. To explain team values in the German Bundesliga, we comprise 15 independent variables. They encompass team information on (1) sporting performance, (2) economic indicators pertaining to the municipality and state, (3) generated revenues, and (4) demographic indicators affiliated with the locality. In these categories, we choose variables that have a clear conceptual connection to team values, are available in our data during the study period, and do not give rise to concerns related to multicollinearity.

Tables 1 and 2 provide descriptive statistics and variable descriptions for all predictor variables. Prior to commencing the analysis, we subjected all predictors, except for categorical variables (dummies), to a scaling process.

**Table 1:** Description of the variables used in the dataset.

Variable	Description
Team_Value	Annual value of the team
TV_Revenue	Annual TV revenue of the team
Win_Percentage	Winning percentage of the team in a season
Goals	Total number of goals of the team per season
Best Eleven	Number of the players in the team of the season
Age	Average age of the team
Total Salary	Total salary of the players in the team
Championships	Total number of championships
Unemployment	Annual unemployment rate of the city
GDP	Gross domestic production per capita of the team city
Population	Total number of population of the team city
Home_Attendence	Average number of home attendence in the stadium
Red Cards	Number of red cards of the team in a year
Yellow Cards	Number of yellow cards of the team in a year

**Table 2:** Descriptive statistics of the variables.

Variable	Observation	Mean	S.D	Min	Max
Team_Value(Million €)	208	172.86	168.34	24.35	948.95
TV_Revenue(Million €)	208	35.96	16.79	9.68	79.6
Win_Percentage	208	40.51	15.45	0.1	85.3
Goals	208	53.61	15.88	25	100
Best Eleven	208	0.64	1.27	0	7
Age	208	25.54	0.9	23.4	27.7
Total Salary(Million €)	208	42.92	39.81	8.1	275.9
Championships	208	4.28	6.53	0	33
Unemployment	208	7.75	3.02	2.8	14.9
Home_Attendence	208	38,994	19,796	15	81,228
GDP(€)	208	41,145	7,489	28,127	69,027
Population (Thousand)	208	761.1	856.2	120	3571
Red Cards	208	2.67	1.72	0	8
Yellow Cards	208	61.07	11.34	34	96

## Modeling

### K-means clustering

The K-means algorithm is an iterative process designed to divide a dataset into K predefined, distinct, non-overlapping subgroups, often referred to as clusters, with each data point belonging to only one cluster. The primary goal is to make the data points within each cluster as similar as possible while ensuring that the clusters themselves are as dissimilar as possible. The algorithm accomplishes this by assigning data points to clusters in a manner that minimizes the sum of the squared distances between the data points and the of their respective clusters. Reducing the variation within clusters results in greater homogeneity among data points within each cluster.

The K-means algorithm employs the Expectation-Maximization approach to address the problem. The Expectation (E)-step involves the assignment of data points to their nearest cluster, while the Maximization (M)-step entails the computation of the centroid for each cluster. Below is the objective function of the K-means:

$$\begin{array}{c} J=\sum_{i=1}^{m}\sum_{k=1}^{K}w_{ik}||x^{i}-\mu_{k}|{|}^{2} \end{array}$$ (1)

For each data point *x*, if it belongs to cluster k, we set *w*_*ik*_ to 1; otherwise, *w*_*ik*_ is assigned a value of 0. Additionally, *μ*_*k*_ represents the centroid of the cluster to which x belongs.

$$\begin{array}{c} \frac{\partial J}{\partial w_{ik}}=\sum_{i=1}^{m}\sum_{k=1}^{K}||x^{i}-\mu_{k}|{|}^{2}\Rightarrow w_{ik}=\{\begin{array}{cc} 1 & k=argmin_{j}||x^{i}-\mu_{k}|{|}^{2}\\ 0 & otherwise \end{array} \end{array}$$ (2)

$$\begin{array}{c} \frac{\partial J}{\partial \mu_{k}}=2\sum_{i=1}^{m}w_{ik}\left(x^{i}-\mu_{k}\right)=0\Rightarrow \mu_{k}=\frac{\sum_{i=1}^{m}w_{ik}x^{i}}{\sum_{i=1}^{m}w_{ik}} \end{array}$$ (3)

$$\begin{array}{c} \frac{1}{m_{k}}\sum_{i=1}^{m_{k}}||x^{i}-\mu_{c^{k}}|{|}^{2} \end{array}$$ (4)

The detailed steps of the k-means clustering algorithm are:

- Determine the desired number of clusters, denoted as K.
- Initialize the centroids by shuffling the dataset and subsequently selecting K data points randomly, ensuring that there is no duplication.
- Continue iterating until the centroids remain unchanged, meaning that the assignment of data points to clusters doesn’t change.
- Calculate the total sum of squared distances between all data points and the centroids.
- Assign each data point to the cluster whose centroid is the closest.
- Compute the new centroids for each cluster by calculating the average of all data points belonging to that cluster.

### Dynamic linear model

We utilize dynamic linear regression models to assess the factors that wield the most substantial influence on team values. Statistical significance is defined as a *p* value below 0.05. The suggested models encompass both fixed and random effects and are as follows:

$$\begin{array}{c} {\text{Y}}_{\text{it}}=\beta_{0}+\beta_{1}X_{1,\text{it}}+\ldots +\beta_{\text{k}}X_{\text{k},\text{it}}+\gamma_{2}{\text{E}}_{2}+\ldots +\gamma_{\text{n}}{\text{E}}_{\text{n}}+u_{\text{it}} \end{array}$$ (5)

In this context, *Y*_*it*_ represents the dependent variable corresponding to team *i* and entity *t*. The independent variables are denoted as *X*_k,it_, with *β*_*k*_ representing the coefficients for these independent variables. The error term is designated as *u*_it_, and *E*_*n*_ signifies the entity *n*, with *γ*_2_ representing the associated coefficient. Furthermore, it is possible to incorporate time effects into the entity effects model:

$$\begin{array}{c} {\text{Y}}_{\text{it}}=\beta_{0}+\beta_{1}X_{1,\text{it}}+\ldots +\beta_{\text{k}}X_{\text{k},\text{it}}+\gamma_{2}{\text{E}}_{2}+\ldots +\gamma_{\text{n}}{\text{E}}_{\text{n}}+\delta_{2}\;{\text{T}}_{2}+\ldots +\delta_{\text{t}}{\text{T}}_{\text{t}}+u_{\text{it}} \end{array}$$ (6)

Where *T*_*t*_ is time as binary variable and *δ*_*t*_ is the coefficient for the binary time regressors. The formulation of the random effect model follows as:

$$\begin{array}{c} Y_{it}=\beta X_{it}+\alpha +u_{it}+\varepsilon_{it} \end{array}$$ (7)

Here, *Y*_*it*_ is the dependent variable, *i* = indicates entity and *t* = indicates time. *X*_k,it_ indicates independent variables, *u*_it_ is the between-error term and *ϵ*_*it*_ is the within-error term.

## Results

### Explanatory data analysis

Our initial approach involves conducting a correlation analysis to explore the relationships between variables and identify potential multicollinearity. To evaluate the level of interdependence among variables, we employed the Pearson Correlation Coefficient. The underlying hypothesis guiding this analysis is as follows: Independent variables should demonstrate a strong correlation with the dependent variable while maintaining a low correlation with each other.

**Fig 1:**
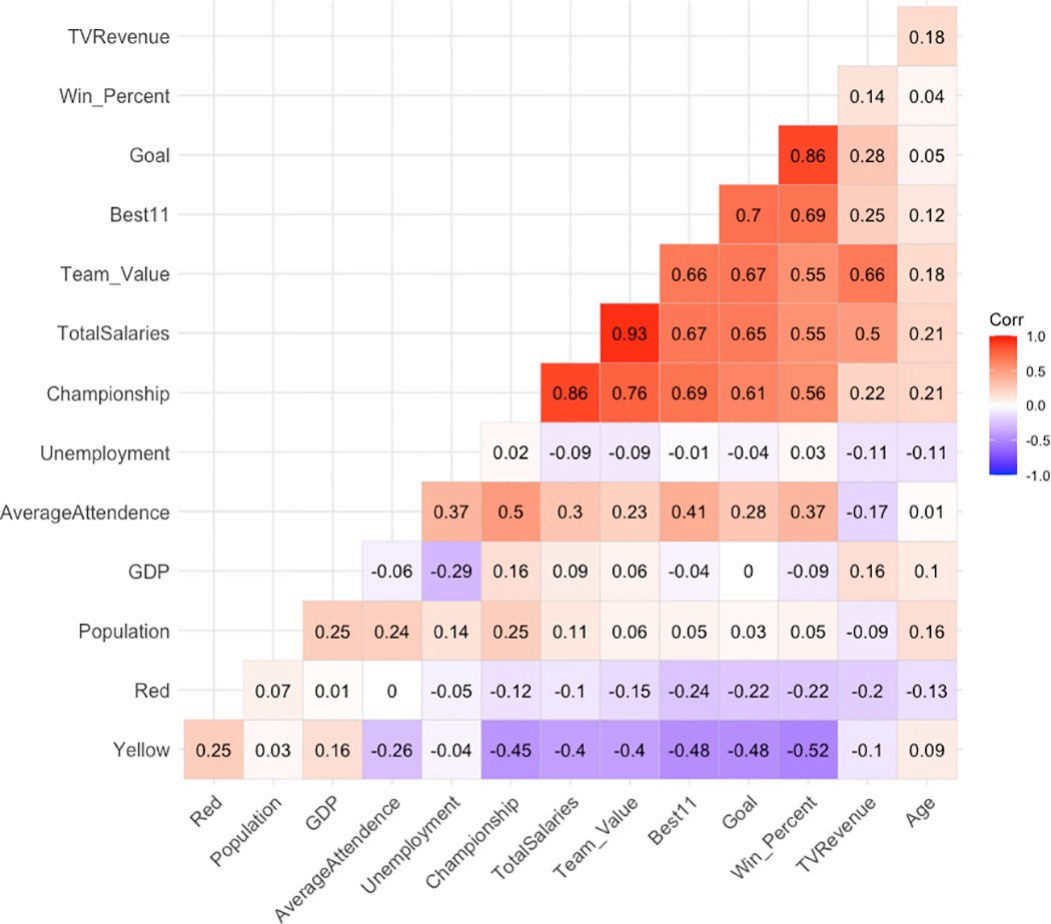
Correlation Plot of the variables.

Fig 1 provides a visual representation of the correlations among all variables, revealing distinct instances of strong correlation. Notably, total salary exhibits a significant positive correlation with championships and other variables (r = 0.86), suggesting that teams with higher revenue levels tend to achieve more championships. Likewise, team value and TV revenue share a robust positive correlation (r = 0.66), indicating that teams with greater value tend to perform better and consequently receive higher shares of TV revenue. On the contrary, there is a negative relationship (r = -0.40) between team value and yellow cards, suggesting that weaker teams may resort more frequently to unfair tactics compared to their stronger counterparts, as noted by [23].

However, it’s worth noting that high correlations can lead to issues of multicollinearity. To assess this, we calculate the Variance Inflation Factor (VIF), and we observed elevated VIF values for Total Salary and Championships, both hovering around 8. In contrast, all other variables exhibit VIFs between 1 and 2. Consequently, we choose to exclude Total Salary from further analysis. To address the issue of serial correlation, we incorporate the lag value of the dependent variable into our models.

In our analysis, we tried to capture and eliminate the interdependencies between variables through correlation analysis. Additionally, we conducted a Principal Component Analysis (PCA). Given that the PCA results aligned closely with the findings from the correlation analysis.

**Fig 2:**
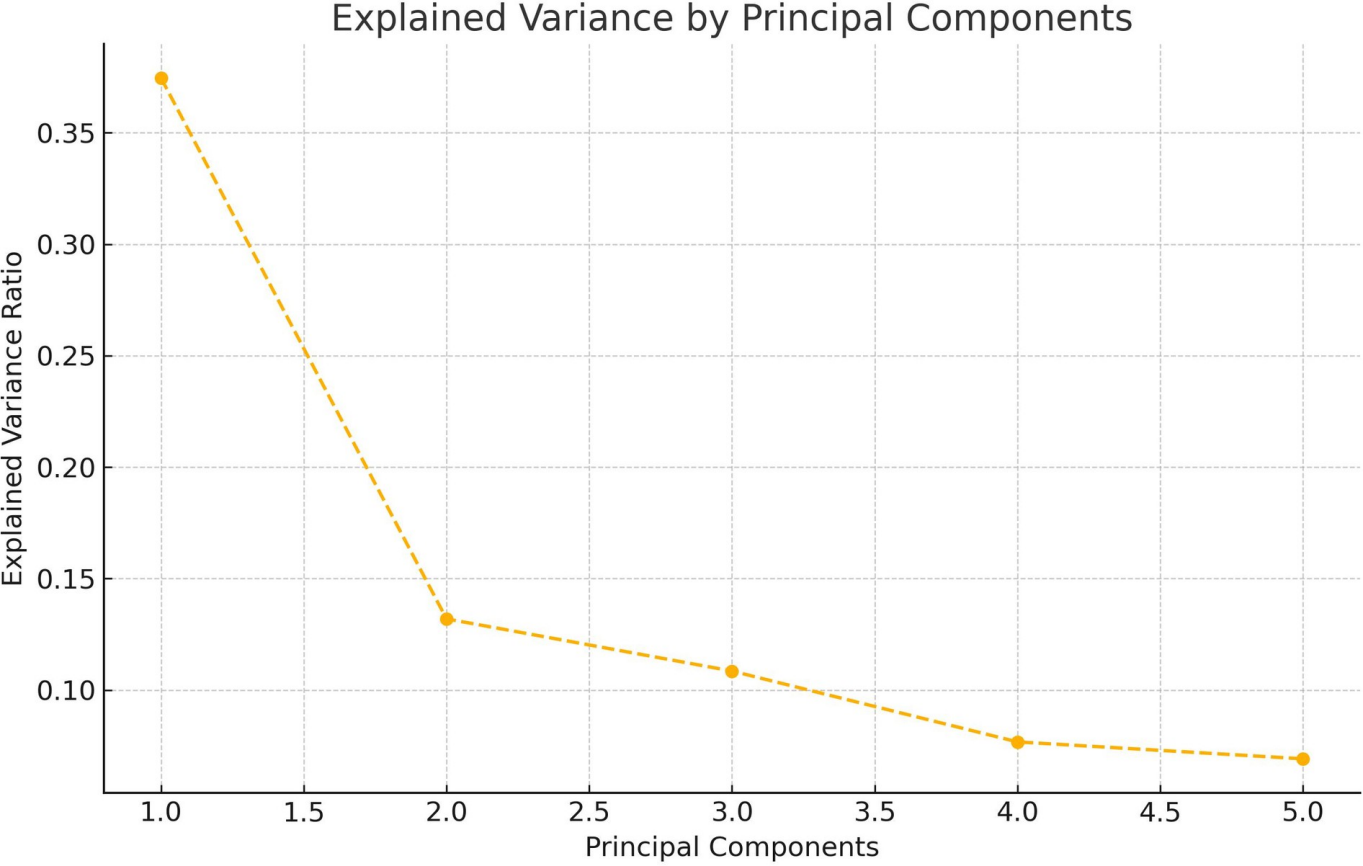
PCA results.

Fig 2 shows the PCA scores obtained from the dataset. PC1 clearly dominates, explaining more than a third of the variance. This means that one major dimension in the data captures a substantial amount of the overall information, likely related to the most influential variables such as team value, TV revenue, and total salaries.

PC2 explains around 13.19% of the variance, meaning it adds a new dimension to the data that is orthogonal (independent) from PC1. This component could relate to secondary factors like demographic or performance-related variables (e.g., win percentage, goals).

PC3, PC4, and PC5 each explain smaller portions of the variance (around 10-7%), representing finer details in the dataset that are less dominant but still relevant.

The results from the PCA are likely reliable and consistent with the findings from the correlation analysis in the paper. The dominance of PC1 aligns with the expectation that certain variables (likely financial ones) are highly correlated and drive much of the variance in the dataset.

**Fig 3:**
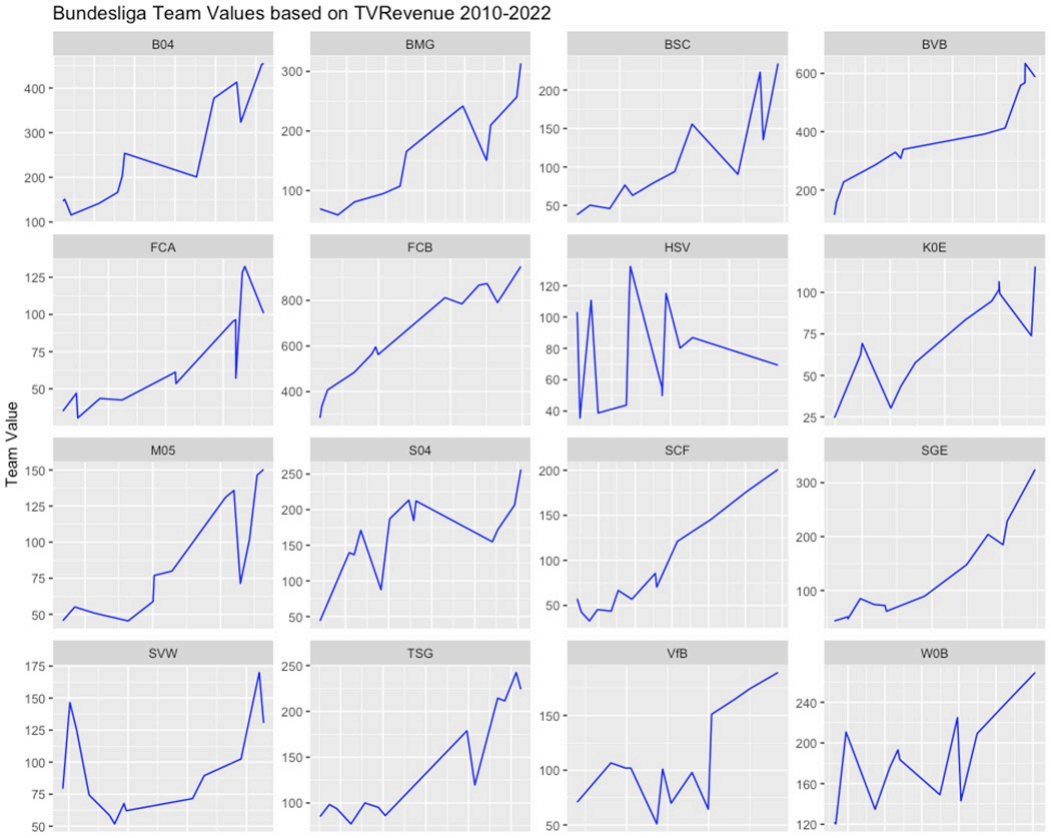
Bundesliga teams value vs TV revenue.

**Fig 4:**
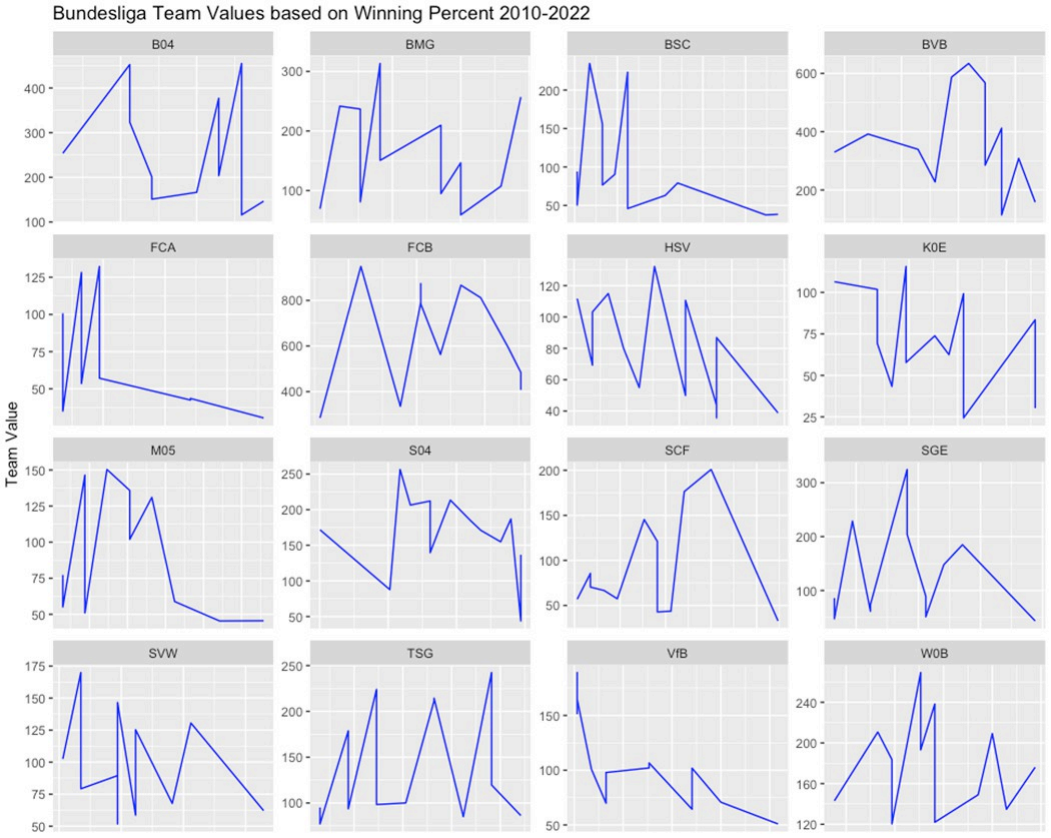
Bundesliga teams value vs winning percentage.

Fig 3 provides a visual representation of the correlation between team values and TV revenue over time, indicating a distinct upward trend. This outcome is expected, given the strong dependency of TV revenues on past team performance. In contrast, Fig 4 offers insight into the dynamic relationship between team value and winning percentage. In this case, the connection between team values and winning percentages appears to be more variable and subject to fluctuations.

Our analysis acknowledges that market value is not solely determined by on-field performance but also by external economic factors such as GDP per capita and revenue structures. Clubs in regions with lower GDP but a history of success can leverage their reputations and success to attract significant investment, enhancing their ability to compete for high-value players. This dynamic creates a complex interplay between economic and performance-based factors in determining player market values.

The results depicted in Fig 4 are quite intriguing. While conventional wisdom might suggest that increasing team value should correspond to higher probabilities of winning, the Bundesliga example challenges this assumption. In this context, it’s evident that as team values increase, the probability of winning tends to decrease. This counterintuitive trend can be attributed to the intensified competition within the Bundesliga. Clubs with lower team values experience more substantial growth in their values over time compared to teams with larger budgets. Consequently, this heightened competition leads to a decrease in the likelihood of winning, making the wining percentages of the champion team from previous years more likely to have lower winning percentages for the following years.

**Fig 5:**
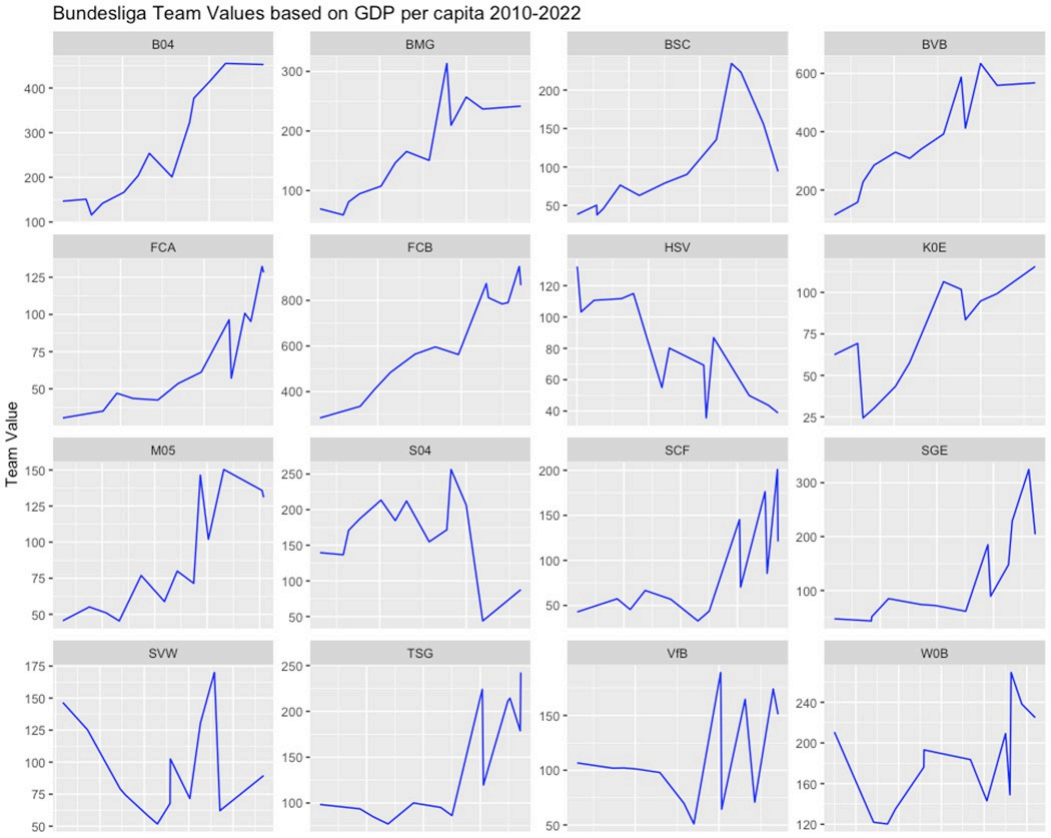
Bundesliga teams values vs GDP.

Fig 5 shows a comparison between team values and GDP for each respective team. While Fig 3 displayed a similar trend, Fig 5 reveals an interesting phenomenon where some team values decreased while GDPs increased. It becomes evident that, for the majority of cities, GDP has a positive impact on team values. When GDP increases, the most team values also experience growth.

### Clustering with machine learning

In machine learning, clustering is an unsupervised learning paradigm employed for the purpose of organizing data points or objects into clusters or groups based on shared attributes. The primary objective of clustering is to unveil latent patterns, structures, or correlations inherent within the data without relying on predefined class labels or categories. It serves as a powerful tool for uncovering patterns and aggregating similar data points. This functionality holds broad relevance across various domains where gaining a deep understanding of the underlying data structure is of utmost significance.

K-Means and Hierarchical Clustering are two widely used techniques in machine learning, utilized to cluster data points based on their similarities. Before venturing into dynamic modelling, we employ these two algorithms to gain a deeper understanding of the data’s characteristics. This approach enables us to discern shared features that contribute to the grouping of teams.

**Fig 6:**
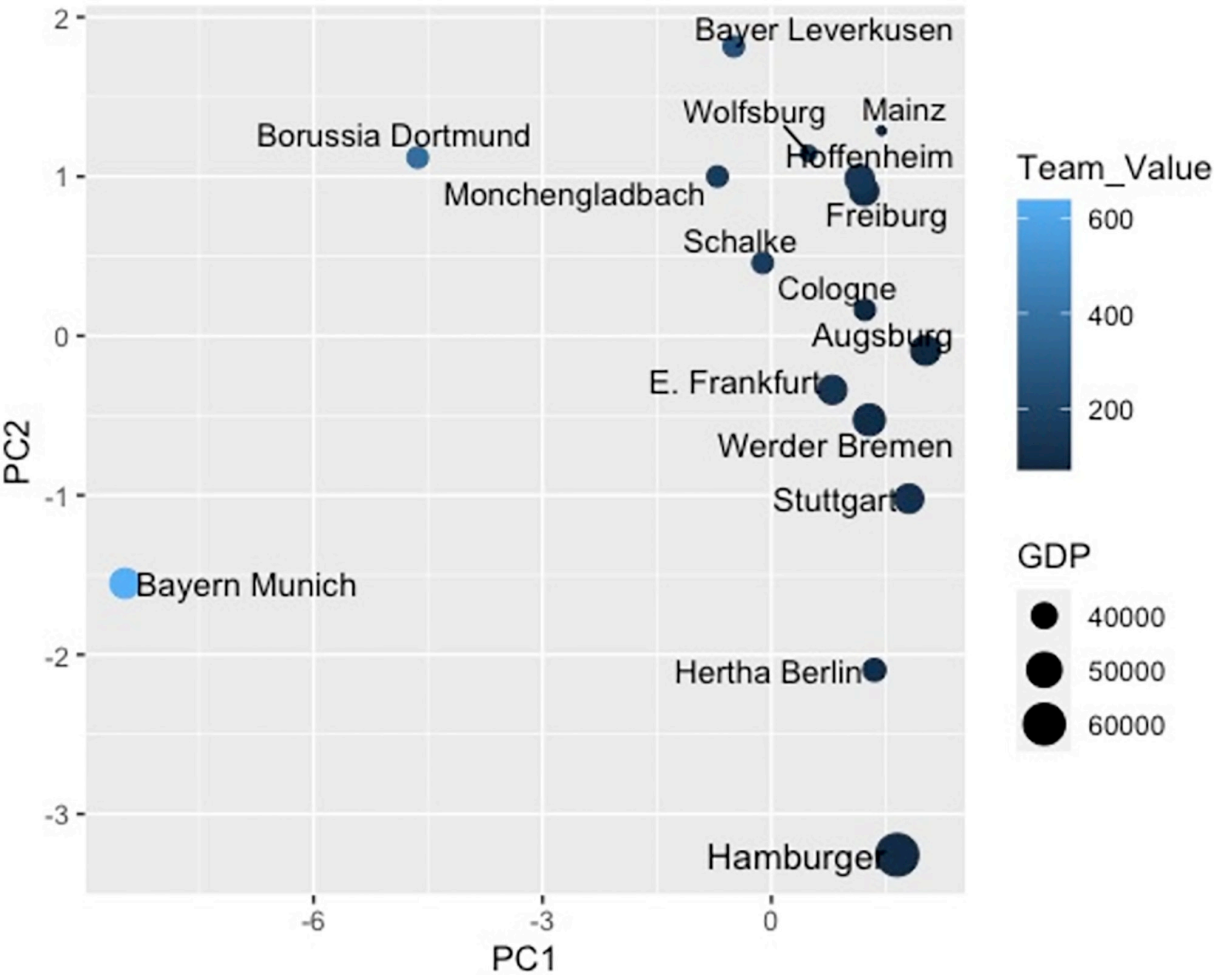
PCA based on team values and GDP.

To begin our exploration, we delve into the clustering of teams by examining the connection between team values and GDP, depicted in Fig 6. Bayern Munich and Borussia Dortmund stand out distinctly from all other teams due to their notably high team values. Additionally, when factoring in GDP, Hertha BSC Berlin and Hamburger SV displayed resemblances, leading to their inclusion within the same cluster. These preliminary findings provide us with initial insights into the team clustering process.

**Fig 7:**
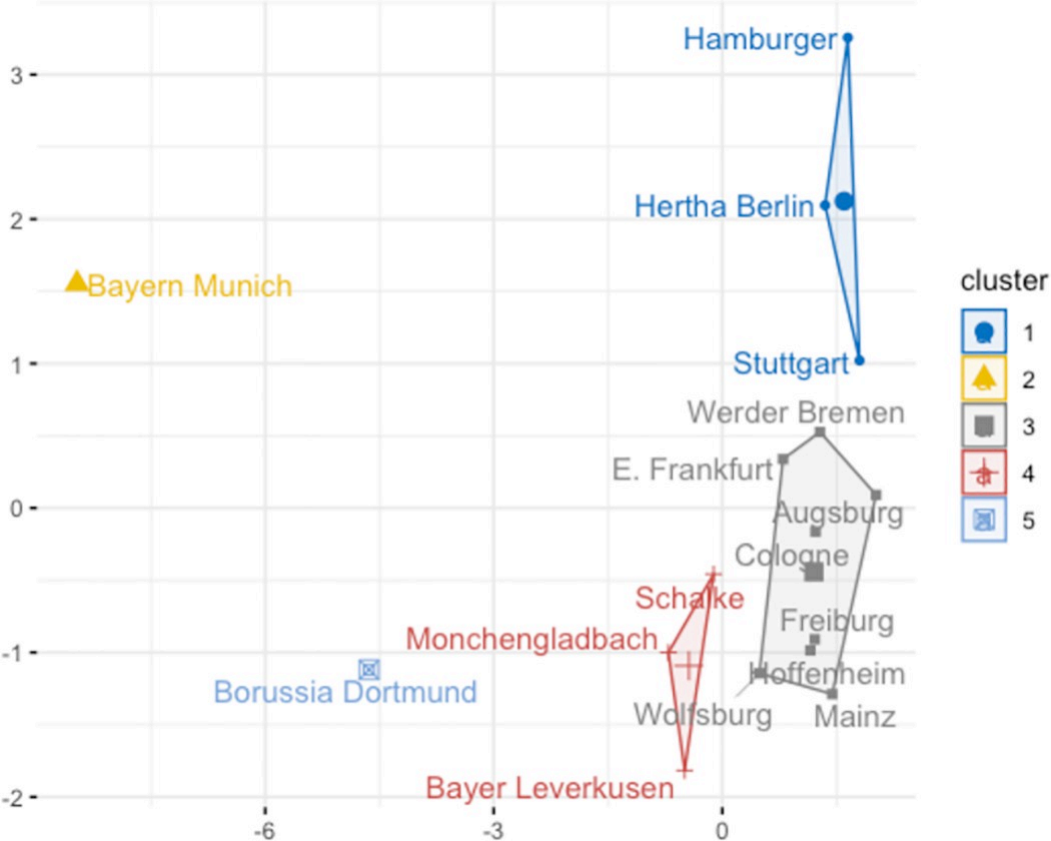
Clustering with k-means algorithm.

The objective of K-Means clustering is to partition data into K clusters, assigning each data point to the cluster with the closest mean (centroid). To determine the ideal number of clusters, we employ gap statistics. Notably, we observe a peak in the gap statistic at k = 5. Therefore, we opt for 5 clusters, aligning with the peak in the gap statistic, and proceed to segment the data into five distinct groups using K-Means clustering. Fig 7 visually represents these clusters, and they can be characterized as follows:

The initial group is denoted by the yellow triangle, and it includes only Bayern Munich. This distinction primarily stems from their extraordinary feat of winning 11 consecutive Bundesliga titles in as many seasons. Their sustained dominance in the league over this time period has set them apart from other teams, granting them the unique position of forming their own distinct group.

The second cluster, represented by a blue square, consists solely of Borussia Dortmund. This exclusivity is attributed to their consistently high team valuation and notable achievements, which prominently distinguish them among Bundesliga teams, trailing only behind Bayern Munich. A scholarly examination of Bundesliga history underscores the logical rationale for categorizing Bayern Munich and Borussia Dortmund into separate groups, given their distinct positions in the league.

The third cluster, symbolized by a red plus symbol, includes Bayer 04 Leverkusen, FC Schalke 04, and Borussia Monchengladbach. A thorough analysis of the teams within this cluster uncovers a noteworthy and intriguing correlation that extends beyond their on-field performance metrics. This correlation is related to the alignment between the television revenue streams generated by these clubs and the Gross Domestic Product (GDP) figures associated with their respective municipalities. The convergence of these economic factors unveils a remarkable parallelism, providing substantial support for the rationale behind their cohesive placement within this specific cluster.

In essence, this alignment underscores the intricate interplay between football clubs and their local economic ecosystems. The television revenue, serving as a critical financial lifeline for these clubs, manifests itself as an economic force with implications extending beyond the boundaries of the football pitch. It reflects not only the popularity and marketability of these clubs on a broader scale but also their contribution to the economic vitality of their respective regions.

The blue circle in Fig 7 represents the formation of another distinct cluster. It encompasses three prominent football clubs with similar historical performances: Hamburger SV, Hertha BSC Berlin, and VfB Stuttgart. Over the span of the 13 seasons considered, all three clubs have undergone the fluctuating fortunes of relegation to lower leagues, only to subsequently regain their status in the prestigious Bundesliga on multiple occasions. These recurring cycles of relegation and promotion highlight the dynamic nature of their footballing trajectories within the German Bundesliga. Given these noticeable criteria, it is reasonable to group the aforementioned trio of teams together.

The final grouping, represented by a grey square, serves as a collective category that encompasses the remaining teams under study. A comprehensive analysis of this assemblage reveals a noticeable convergence in average team values. However, it’s important to note exceptions in the form of Eintracht Frankfurt, VfL Wolfsburg, and TSG 1899 Hoffenheim, which exhibit discernible deviations from this closely clustered norm. Moreover, it is imperative to acknowledge that the clubs constituting the grey square grouping, exhibit an additional dimension of similarity through their on-field achievements, which surpass the statistical mean of success performances within the league. The resonance of their success metrics above the league average accentuates the robustness of their competitive prowess, making them an integral part of this amalgamated group.

The clustering derived through machine learning methodologies serves as valuable guidance in elucidating the distinctive configurations of teams and the intricate architecture essential for subsequent modelling endeavours. It is particularly instrumental in discerning the manner in which various attributes are aggregated into analogous clusters. Consequently, these findings have proven to be instrumental in enhancing our comprehension of the variable components within the dynamic linear model that is slated for development.

**Fig 8:**
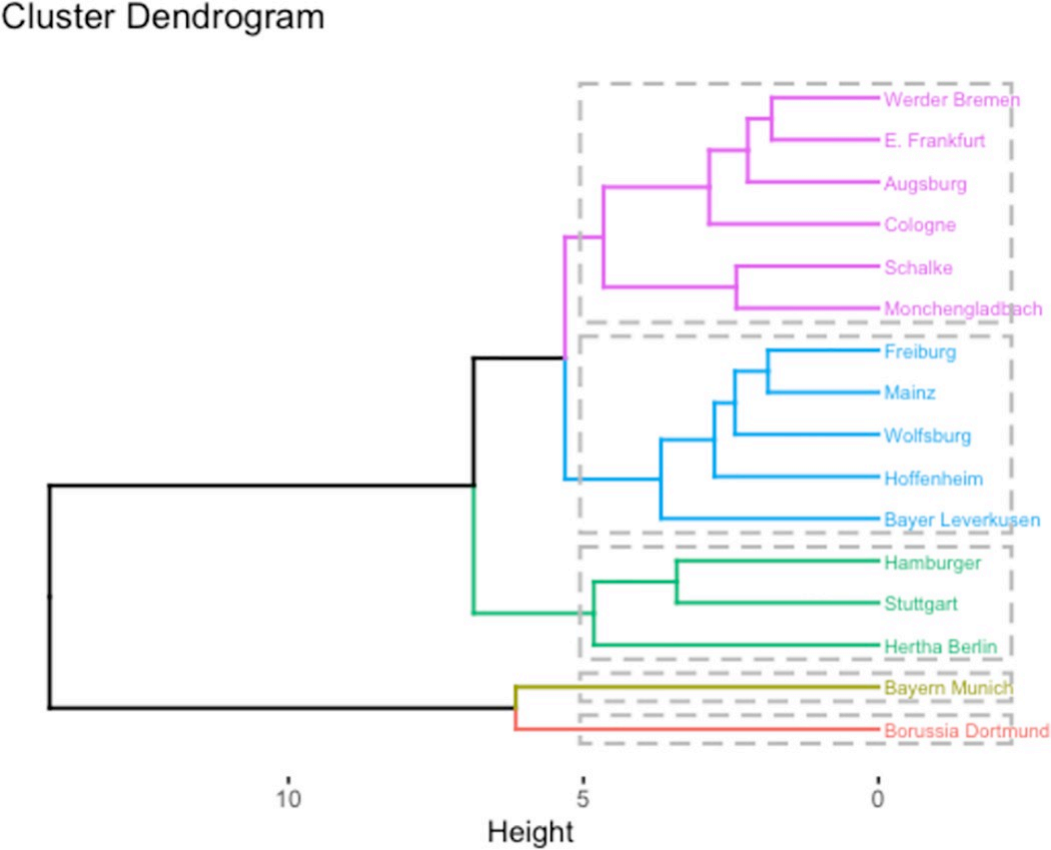
Plot of hierarchical clustering.

The results obtained from hierarchical clustering align closely with those generated by the K-means clustering approach which can be seen in Fig 8. Nevertheless, it is noteworthy that some teams displayed variations in their cluster assignments. This discrepancy can be attributed to the positioning of these teams on the fringes of the cluster boundaries. Consequently, it is apparent that different clustering techniques may assign certain teams to alternative clusters based on their placement within the dendrogram.

### Dynamic linear models

The synthesis of dynamic linear models is presented in Table 4, encompassing a comprehensive array of seven distinct modelling paradigms, namely Ordinary Least Squares (OLS), Fixed Effect Model, Linear Fixed Effect Model, Dummy Variable Fixed Effect Model, Random Effect Model, Linear Random Effect Model, and Dummy Variable Random Effect Model. Within Table 4, significance levels are denoted by asterisks (*), with significance at the 0.1 level, circles • signifying significance at the 0.05 level, and daggers † indicating significance at the more stringent 0.01 level.

The variables encompass inherent team characteristics. Consequently, conventional Ordinary Least Squares (OLS) models manifest inherent limitations in effectively addressing this information. Conversely, fixed effects models are adept at probing the intricate interplay between dependent and independent variables within individual entities, with each entity characterized by distinctive attributes that wield influence upon the dependent variables. In this regard, fixed effects models stand out as a robust and effective solution to address this particular challenge.

On the other hand, random effects models suggest that the variations observed both across and within entities are inherently stochastic, showing no discernible correlation with either the dependent or independent variables. As a result, we contend that fixed effects models are better positioned to provide reliable estimations than other dynamic models.

Table 3 features the results of several models, predicated upon their respective Akaike Information Criterion (AIC) and R-squared (*R*^2^) scores. From this rigorous evaluation, the model that attains the highest in *R*^2^ scores and the lowest in AIC scores is selected as the preeminent model. It becomes evident from Table 3 that the fixed effects model featuring dummy variables exhibits the most favourable combination of the highest *R*^2^ score and the lowest AIC score.

**Table 3:** Comparison of dynamic linear models.

Independent Variables	Dependent Variable: Team_Value						
	OLS	FE	Linear FE	Dummy FE	RE	Linear RE	Dummy RE
TV_Revenue	6.340† (0.580)	2.831†(0.313)	3.133†(0.451)	4.009† (0.490)	2.968† (0.388)	3.016† (0.450)	4.032†(0.496)
Win_Percentage	0.598 (0.626)	0.472(0.488)	0.414(0.492)	0.265(0.483)	0.118 (0.477)	0.118 (0.478)	0.174 (0.468)
Best11	0.383 (5.787)	-1.407 (4.611)	-1.234 (4.617)	-2.152(4.456)	-3.404 (4.509)	-3.428 (4.522)	-4.079(4.323)
Age	-7.138(4.914)	-2.415(4.105)	-2.325(4.107)	-3.185(4.008)	2.083(4.215)	2.128(4.232)	1.180(4.141)
Championships	13.9† (1.128)	3.239*(1.657)	3.207*(1.656)	2.859*(1.757)	9.263*(5.409)	9.1*(5.478)	9.822*(5.343)
Unemployment	-3.831•(1.869)	-0.419(1.668)	-0.335(1.668)	-1.882(1.889)	1.553(6.107)	2.129(6.705)	-3.680(7.228)
Home_Attendence	0.0002(0.0005)	0.0001(0.0002)	0.0001(0.0002)	0.0002(0.0004)	0.0003(0.0003)	0.0003(0.0003)	0.001(0.001)
GDP	0.001*(0.001)	-0.001(0.001)	0.001(0.001)	0.005*(0.001)	0.004*(0.002)	0.003* (0.004)	0.001(0.004)
Population	0.004(0.005)	0.002(0.006)	0.002(0.006)	0.002(0.006)	-0.438• (0.209)	-0.431• (0.213)	-0.466•(0.209)
Red	4.236 (2.599)	1.406(2.039)	1.108 (2.066)	0.716(2.008)	1.109(1.956)	1.051(1.980)	0.523(1.918)
Yellow	-0.068 (0.471)	-0.015(0.377)	0.055(0.385)	-0.313(0.382)	0.331(0ç388)	0.345(0.395)	-0.009(0.389)
Goals	1.502 • (0.599)	0.954 •(0.467)	0.971• (0.468)	0.679 (0.467)	0.653 (0.453)	0.650 (0.454)	0.248 (0.456)
Season			-1.743(1.876)				
Season 11				-7.237 (15.353)			
Season 12				-10.429(15.469)			
Season 13				-27.048*(15.868)			
Season 14				-11.852(16.519)			
Season 15				-20.688(16.983)			
Season 16				-42.096• (17.584)			
Season 17				-86.128† (21.257)			
Season 18				-71.886† (22.602)			
Season 19				-48.326• (23.062)			
Season 20				-45.501(28.701)			
Season 21				-41.115* (24.039)			
Season 22				-38.214 (24.030)			
AIC	2299.58	2190.01	2191.23	2180.43	2239.81	2241.76	2228.94
Observations	208	208	208	208	208	208	208
*R* ^2^	0.899	0.896	0.897	0.907	0.805	0.805	0.835
Adjusted *R*^2^	0.885	0.889	0.889	0.893	0.773	0.772	0.794

*: Statistically significant at a significance threshold of 0.1,

^•^: Statistically significant at a significance threshold of 0.05,

^†^: Statistically significant at a significance threshold of 0.01

The extended analytical purview features lagged dependent variables, enhancing the robustness of our estimates pertaining to the impacts of the independent variables. Within this analytical framework, we delineate two distinct levels of lagged dependent variables as integral components of the model. This methodological nuance was implemented to effectively account for and mitigate auto-correlation effects intrinsic to the error term.

As shown in Table 4, we employed fixed effects models incorporating one and two lagged dependent variables against the winning model. Including temporal lags for team value within the model context not only aids in unravelling the intricate variations in team value during a specific time period but also significantly bolsters the accuracy of our parameter estimations. This inclusion of lagged variables serves as a pivotal mechanism for yielding more precise parameter estimates, thus reinforcing the analytical robustness of our study.

**Table 4:** Comparison of fixed effects models.

Independent Variables	Dependent variable Team_Value		
	Dummy FE	Dummy FE(lag = 1)	Dummy FE(lag = 2)
Team_Value(lag = 1)		0.662† (0.047)	0.200 † (0.075)
Team_Value(lag = 2)			0.126(0.080)
TV_Revenue	4.009† (0.490)	2.734† (0.459)	3.110† (0.468)
Win_Percentage	0.265(0.483)	0.149(0.433)	0.144 (0.440)
Goals	0.679(0.467)	0.887• (0.412)	0.273 (0.427)
Best11	-2.152 (4.456)	-3.954 (3.964)	-4.173 (4.380)
Age	-3.185(4.008)	-2.259(3.470)	-2.720(3.881)
Championships	2.859(1.757)	3.872† (1.043)	0.676 (5.127)
Unemployment	-1.882 (1.889)	-2.933• (1.294)	-7.590(7.672)
Home_Attendence	0.0002 (0.0004)	0.0004 (0.0003)	0.0004 (0.0005)
GDP	0.005*(0.001)	0.004*(0.001)	0.001 (0.004)
Population	0.002 (0.006)	0.003(0.004)	-0.375*(0.220)
Red	0.716(2.008)	1.105(1.830)	0.218 (1.880)
Yellow	-0.313(0.382)	-0.336(0.328)	-0.403 (0.357)
Season 11	-7.237 (15.353)		
Season 12	-10.429(15.469)		
Season 13	-27.048*(15.868)		
Season 14	-11.852(16.519)		
Season 15	-20.688(16.983)		
Season 16	-42.096•(17.584)		
Season 17	-86.128†(21.257)		
Season 18	-71.886†(22.602)		
Season 19	-48.326• (23.062)		
Season 20	-45.501(28.701)		
Season 21	-41.115*(24.039)		
Season 22	-38.214(24.030)		
AIC	2191.23	1962.85	2237.9
Observations	208	192	176
*R* ^2^	0.907	0.958	0.864
Adjusted *R*^2^	0.893	0.951	0.822

*: Statistically significant at a significance threshold of 0.1,

^•^: Statistically significant at a significance threshold of 0.05,

^†^: Statistically significant at a significance threshold of 0.01

Based on the findings presented in Table 3, we have identified the fixed-effects model augmented with dummy variables and a lag parameter of one (lag = 1) as the preferred and conclusive model. It is imperative to emphasize that within the final model, a total of five variables—TV Revenue, Unemployment, GDP, Championships, and Goals—manifest statistically significant associations with the dependent variable, thereby underscoring their substantive relevance within the analytical framework.

Based on the conclusive findings from the winning model, particularly when assessing team values with a lag parameter of one (lag = 1), it is evident that TV Revenue, GDP, Championships, and Goals all exert a substantial and statistically significant positive influence on team value, while unemployment shows a negative impact. Notably, the coefficients associated with Championships and TV Revenue stand out as particularly significant, highlighting their pronounced importance in the dynamics of team value. For instance, it is observed that for every championship secured, the team’s value experiences an approximate increase of €3.9 million. This empirical insight underscores the pivotal role played by championship victories in enhancing team value. Furthermore, an increase of €1 million in TV revenue is associated with a rise of approximately €2.734 million in team value, underscoring the profound impact of television revenue streams on a team’s overall worth.

Notably, Bayern Munich and Borussia Dortmund consistently demonstrate the highest average team values and boast the most substantial TV revenue earnings. The empirical evidence strongly supports the hypothesis suggesting that teams with robust TV revenue streams and a history of championship victories exhibit correspondingly higher team values. When considering statistical significance, it’s worth highlighting that both unemployment and the Goals variables achieve statistical significance at the *α* = 0.05 significance level. Particularly, unemployment displays a detrimental effect, where a one percent increase in unemployment corresponds to a decrease of approximately €2.9 million in team value. On the other hand, GDP, though statistically significant at the *α* = 0.1 significance level, demonstrates a notable positive association. This indicates that each €1,000 increment in GDP is associated with a €4 million increase in team value. These findings collectively underscore the substantial impact of economic indicators compared to performance-related variables when estimating team value. It becomes apparent that team value is profoundly influenced by economic determinants, including TV revenue, unemployment, and GDP.

## Discussion

The primary objective of this research was to closely examine the economic indicators and performance variables that impact the valuation of Bundesliga teams. To achieve this objective, we employed sophisticated methodologies, specifically machine learning clustering techniques and dynamic linear models, to conduct a thorough and comprehensive analysis.

In our initial correlation analysis, we observed a significant correlation between total team salaries and several other predictors. Consequently, we excluded total salary from subsequent analyses. Additionally, we identified a strong positive correlation between Gross Domestic Product (GDP) and the team values. Conversely, winning percentage and the frequency of yellow cards correlated negatively, suggesting that a higher number of yellow cards adversely affects a team’s winning percentage. As expected, we found that television (TV) revenue has a positive impact on team valuation. These insights were subsequently integrated into the modeling process to inform the subsequent analytical phases.

In our cluster analysis, we employed both K-means and hierarchical clustering techniques to assess teams in terms of their overall influence. We accounted for a total of 13 distinct variables. Utilizing a distance matrix, we categorized teams into separate groups to unveil the key traits of their overall impact. Our analysis determined an ideal cluster count (k) of 5, as indicated by the gap statistic. Subsequently, we divided the data into five clusters through K-means clustering and proceeded with further analysis accordingly. Hierarchical clustering likewise resulted in a five-cluster classification.

In modeling team values, we delved into dynamic regression models, comparing seven different models based on criteria such as the Akaike Information Criterion (AIC) and R-squared (*R*^2^) scores. Our analysis revealed that the optimal choice in the initial assessment was the dummy variable fixed effects model. Across all the models considered, TV revenue consistently emerged as the most important influence of team values. This observation aligns with findings from various studies, which highlight the positive relationship between TV revenue and team valuations. For instance, [24] demonstrated that TV revenue plays a pivotal role in determining club valuations in the English Premier League. This trend is not limited to the Premier League, as similar results have been reported in other leagues, including the Bundesliga and La Liga [25].

Subsequently, we expanded our analysis to incorporate models that included lagged dependent variables, aiming to provide robust estimates of the effects of independent variables. The final selected model was the dummy fixed effects model with a lag of 1. According to this model, TV revenue, GDP, Championship titles, and Goals each demonstrated a statistically significant positive impact on team values, while Unemployment showed a negative effect. Research conducted by [16] suggests that local economic conditions can indeed influence the valuation of football clubs. Our findings within the context of the Bundesliga support the notion that economic factors play a significant role in team valuations. Notably, the coefficients associated with Championship titles and TV revenue show strong significance in shaping team valuation, indicating that teams with a greater number of championships, higher GDP, and increased TV revenues tend to command higher valuations. Research by [26] on Belgian football clubs also shows that sporting success, such as winning league titles, positively affects club valuations, which aligns with the conclusions of our study.

In addition to economic variables, we examined performance-related factors and observed that specific elements, including red cards, yellow cards, and winning percentages, did not yield statistically significant effects on team valuation. These results contrast with findings from earlier research. It is conceivable that the driving forces behind team valuation primarily stem from indicators such as TV revenue and championship titles, among others.

It is crucial to acknowledge the presence of additional variables that could exert an influence on team valuation, such as advertising agreements, sponsorships, and stadium advertising. Future research should take into account the inclusion of these factors, notwithstanding the inherent difficulties associated with procuring such data due to cost and limited accessibility. Nevertheless, the endeavor to acquire and integrate these variables is imperative, as they have the potential to fluctuate significantly between teams and exert a notable influence on team valuations.

Since analyzing such economic datas are ver complicated, there are some limitations of the study. One key limitation is the availability and granularity of the data. Although we have used extensive data across multiple seasons, certain variables, such as detailed revenue breakdowns (e.g., sponsorship, merchandise, and matchday revenues), were not available at the desired level of granularity. This lack of detailed data may limit the precision of our conclusions about the full impact of various revenue streams on market values and personnel expenditures. Furthermore, there are potential unobserved variables, such as managerial quality, team cohesion, or other strategic factors, that could influence both market values and expenditures. These factors were not explicitly included in our model, which may limit our ability to fully capture all influences on team performance and market value.

Finally, while the findings are specific to the Bundesliga and reflect dynamics within this league, they may not be fully generalizable to other leagues with different revenue distribution models, competitive structures, or regulatory environments. Future studies could benefit from comparing these dynamics across multiple leagues to determine the extent to which our findings hold in different contexts.

## Conclusion

Our findings provide valuable insights into German Bundesliga team values, offering a comprehensive analysis that combines correlation analysis, clustering, and linear modeling to uncover meaningful patterns and relationships within the data. Remarkably, within the spectrum of all participating teams, the cluster analysis results evinced an exceptional degree of prominence in the case of Bayern Munich, setting it apart distinctly from its peers. Borussia Dortmund is the second one-team-cluster in our data. Moreover, this analytical approach enabled us to explicate the underlying rationales for the comparatively low team valuations witnessed among select Bundesliga teams domiciled in densely populated urban centers. Within the ambit of the cluster analysis, Hamburger SV and Hertha BSC Berlin feature a third cluster, both of which represent teams hailing from highly populous German cities. Notably, both teams concurrently exhibited the most modest average team valuations within the Bundesliga. This empirical evidence underscores the indispensability of incorporating economic parameters such as Gross Domestic Product (GDP) and TV revenue into the predictive modeling of team valuations within the Bundesliga framework. The next cluster encompassed teams characterized by average performance metrics, notably lacking in championship accolades over the preceding three decades. Consequently, a majority of these teams exhibited middling team valuations.

Concluding, this study underscores the pivotal role of specific economic variables and demographic indicators in the determination of team valuations, i.e., TV revenue, unemployment rates, championship history, GDP, and goals performance. An important outcome of this study is the realization that team valuation is not exclusively contingent upon performance-related statistics; instead, ancillary factors such as GDP and the unemployment rate within the team’s urban domicile should be accorded due consideration when conducting team valuation analyses. It is crucial to refrain from exclusive reliance upon performance-driven variables when undertaking team valuation analyses, given the potential for misleading outcomes. Finally, this study aims to underscore the importance of analyzing economic factors alongside team valuations. The findings of this research carry significant implications for team owners and managers. Our model provides a crucial tool that can assist managers and owners in developing strategies to enhance team valuations, making them better equipped to thrive in various competitive scenarios.
